# Yolk metabolomics reveals candidate compounds associated with egg specific density and hatchability in white layer breeder hens^☆^

**DOI:** 10.1016/j.psj.2026.107356

**Published:** 2026-06-29

**Authors:** Thomas A.C. Calil, Bruno S. Vieira, Simone Sommerfeld, Arlene B. dos S. Nossol, Tiara da C. Silva, Douglas Q. Santos, Ana Clara Feitosa Silva, Mário M. Martins, Victor Aguiar de Souza Penha, Mauricio Cunha Escarpinati, Hebréia Oliveira Almeida Souza, Marcelo E. Beletti, Anna Luiza F.D. Strack, Belchiolina Beatriz Fonseca

**Affiliations:** a Federal University of Uberlândia, Uberlândia, Minas Gerais, Brazil; b Federal University of Triângulo Mineiro, Uberaba, Minas Gerais, Brasil; c Department of Integrative Biology, Michigan State University, East Lansing, Michigan, USA

**Keywords:** Specific density, Hatchability, Biomarkers

## Abstract

The present study aimed to evaluate the influence of egg geometry, weight loss, and specific density (SD) on hatchability in older white layer breeder hens. In addition, based on the positive correlation observed between fertility and egg SD, we hypothesized that the mineral and metabolomic composition of the yolk may be associated with specific density. A total of 8,874 eggs from five Lohmann LSL Lite breeder flocks (52–64 weeks) were analyzed. We found that digital image analysis is an adequate method for evaluating the egg shape index, whereas Archimedes’ principle is suitable for determining SD. Although we observed an influence of shape index and weight loss on hatchability, the clearest findings were related to SD. Eggs within the upper 50% SD range (1075-1110g/L) exhibited higher hatchability than those with lower SD, primarily due to reduced infertility and lower early embryonic mortality. Low-SD eggs showed increased contamination rates. Yolk mineral concentrations did not differ significantly between SD groups, except for the Na:K ratio. However, the N;Ka ratio showed minimal predictive value, indicating that mineral composition alone has limited explanatory power for eggshell density. Untargeted metabolomics identified 310 metabolites in yolk, but only melatonin glucuronide and dihydroxytetradecanoic acid met the significance criteria (p<0.05; fold change > 2). This study provides a precise way to evaluate egg geometry and SD. Furthermore, using a large dataset, it demonstrates that eggs with low SD have reduced hatchability due to infertility and early mortality, likely linked to contamination. Although the Na:K ratio, melatonin glucuronide, and dihydroxytetradecanoic acid showed limited predictive performance when evaluated individually, their identification highlights potential biochemical differences associated with SD and supports their further investigation as candidate markers.

## Introductions

In the poultry industry, hatchability is a key trait because it directly influences chick production (Narushin et al., 2021; Oliveira et al., 2023). The egg is a biological system specifically designed to ensure both embryo protection and successful hatching; therefore, hatchability is strongly influenced by the quality of the fertile egg (Alkan et al., 2015; Chen et al., 2024; Oliveira et al., 2023; Reijrink et al., 2009; Wolc et al., 2010). The physical and external characteristics of the egg, such as egg mass, shell thickness, shape index, and porosity, are closely linked to successful or unsuccessful hatchability (Alkan et al., 2015; Fathi et al., 2022; Narushin et al., 2021).

Eggshell thickness and porosity regulate carbon dioxide and oxygen internal concentrations during embryo development. Shell thickness may correlate with moisture loss during the incubation period. In the poultry industry, thick-shelled eggs tend to experience less moisture loss than thin-shelled ones, resulting in better hatchability (Fathi et al., 2022; Malik et al., 2015; Roque and Soares, 1994). Moreover, the shell serves as a protective barrier against microbial contamination and mechanical damage (Malik et al., 2015). To assess shell thickness and determine its influence on hatchability, measuring egg specific density (SD) provides an effective approach. Specifically, among the methodologies to measure SD, Archimedes’ principle can be employed to evaluate shell quality with higher accuracy (Karabulut, 2021).

Another factor influencing hatchability is the morphological shape of the egg. Eggs with normal morphological shapes have a greater likelihood of successful hatching compared with deformed eggs (Malik et al., 2015). More recently, photographic imaging techniques have been employed to gather information on egg morphology (Adegbenjo et al., 2020; Yudhana and Saifullah, 2017). Assuming that incubation involves exchanges between the inside and outside of the egg and its surrounding environment, we hypothesized that changes in the shell surface related to the egg’s volume could contribute to differences in incubation results.

There is a positive association between SD, hatchability, and fertility (Karabulut, 2021; Segura et al., 2024). However, the biological basis underlying this relationship remains poorly understood. Egg SD is commonly interpreted as an indirect indicator of eggshell quality and mineralization, and the eggshell plays a critical role as a physical barrier against water loss and microbial penetration, while also supporting gas exchange during incubation (Nys and Guyot, 2011; Tullett and Burton, 1985). Eggshell formation depends on a highly regulated calcification process in the shell gland, involving calcium supply, bicarbonate availability, ion transport, and matrix-mediated calcium carbonate deposition (Arad et al., 1989; Jonchère et al., 2012; Nys and Guyot, 2011).

In parallel, the yolk is the main nutritional reservoir for the developing embryo, providing lipids, proteins, minerals, and bioactive compounds required for early embryonic metabolism and development (Tian et al., 2023; Wood et al., 2021). Minerals are essential for fertility (Yaripour et al., 2018) and embryonic development (Uni et al., 2012), contributing to osmotic balance, enzyme activity, tissue development, and early embryo physiology. In addition, yolk metabolites may reflect maternal nutrient allocation and biochemical pathways associated with embryo viability (Meng et al., 2021). Metabolomics can identify new metabolites or changes in the proportion of low-molecular-weight compounds, making it a useful approach to investigate the physiological association between shell quality, yolk composition, and hatchability. Therefore, we hypothesized that eggs with different SD would also differ in yolk mineral and metabolomic profiles, potentially helping to explain differences in fertility, early embryonic mortality, and hatchability.

Studies on egg quality related to shape index and SD are essential for a deeper understanding of the relationships and mechanisms that influence hatchability. Specifically, the relationship between yolk composition and eggshell quality is underexplored in scientific literature. This type of study is crucial for understanding whether components in the yolk are associated with eggshell quality and for investigating potential factors that could explain the positive relationship between SD and fertility. The present study aimed to evaluate the influence of egg geometry, weight loss, and SD on hatchability, as well as to determine whether the mineral and metabolomic composition of the yolk interferes with egg SD.

## Materials and methods

### Birds and eggs

A total of 8,874 eggs from five Lohmann LSL Lite breeder flocks aged between 52 and 64 weeks were evaluated. These flocks were classified by the egg-providing company as old breeder flocks, a category in which changes in egg quality, shell characteristics, and hatchability are more likely to occur (Lohmann Tierzucht, 2018). A total of 8,874 eggs from five Lohmann LSL Lite breeder flocks aged between 52 and 64 weeks were evaluated. These flocks were classified by the egg-providing company as old breeder flocks, a category in which changes in egg quality, shell characteristics, and hatchability are more likely to occur. The sample size was defined based on the number of eggs available from eligible flocks and on the total number of eggs that could be processed during the collection period, considering the logistical and operational capacity of the study. This approach allowed the generation of a large-scale phenotypic dataset to assess associations among egg geometry, weight loss, specific density, and hatchability outcomes.

Furthermore, working with a single flock age group would avoid the influence of different diets supplied to the birds, which could affect egg parameters. The eggs used were between 2 and 3 days post-lay, stored at 20°C ± 1°C (Lohmann Tierzucht Hatchery Guide, 2018), and subjected to the same standard procedures adopted by the supplying company for dry disinfection on the farm and in the hatchery. The breeders' nutrition remained the same throughout the study period, with the nutritional levels used described in Supplementary Table 1.

This experiment was conducted in four distinct analyses. First, we performed preliminary tests to evaluate whether Archimedes' principle is an adequate methodology for assessing SD. Next, we performed digital image measurements of the egg’s geometric characteristics. Subsequently, we analyzed the factors affecting hatchability. Finally, after observing the importance of SD for hatchery success and fertility, we evaluated the relationship between SD and yolk components.

Both the rearing and production of all flocks were carried out in environmentally controlled houses with automatic nests in the city of Comendador Gomes, Triângulo Mineiro region, Minas Gerais, Brazil, using a full-slat configuration in the production barns. During the breeding and rearing phases, the birds were vaccinated with live and inactivated vaccines against Marek’s disease, Gumboro disease, Newcastle disease, infectious bronchitis, metapneumovirus, infectious anemia, encephalomyelitis, as well as with a recombinant vaccine against laryngotracheitis. During the production phase, the birds were vaccinated with a live vaccine against infectious bronchitis at 46, 52, 58, and 65 weeks of age. In weeks when vaccinations were performed, eggs were collected prior to the vaccination procedure.

Following disinfection at the hatchery (2.5g formaldehyde/m3 for 30 minutes, including exhaust), eggs were randomly selected before grading, always from adjacent rows of trays placed on transport carts.

At hatch, the chicks were subjected to the company’s standard hatchery procedures and subsequently transported to the production farm. No additional handling was performed beyond routine hatchery practices. The study was certified by the Committee on Ethics in the Use of Animals (CEUA) of the Federal University of Uberlândia under approval number A001/21.

### Preliminary tests

We performed preliminary tests to evaluate the feasibility of Archimedes' methodology for SD measurement. Archimedes' method may offer advantages over saline solution for evaluating egg SD due to its higher precision and reproducibility (Calil, 2023; Thompson and Hamilton, 1982).

A total of 2,318 eggs from two flocks were analyzed by immersion in a saline solution (Oliveira and Oliveira, 2013), using categories of 1055, 1065, 1075, 1080, 1085, and 1095g/L, for subsequent comparison and concordance testing with the Archimedes’ principle methodology. For the Archimedes’ principle analysis (Hamilton, 1982; Hempe et al., 1988), the same eggs were tested. Each egg was weighed on a digital scale (Maxx SK303; 3kg capacity; 0.1g precision), then suspended using strings and a plastic egg cup on a structure positioned above a water container, submerged in water at 20°C, and weighed again on the same scale. The final density value was multiplied by a correction factor of 0.998 to account for water density at 20°C, according to (Kell, 1975). The buoyancy force of the container and structures supporting the egg in the water was disregarded, as the scale was tared with this apparatus already submerged, as mentioned by (Hempe et al., 1988) (Supplementary Figure 1). The ratio between the dry and submerged weights, multiplied by the water density correction factor at the applied temperature, results in the egg density, expressed in grams per liter.

As the Archimedes' principle had similar average results to the saline method, we performed additional tests to evaluate whether Archimedes' principle reflects shell quality regardless of yolk and albumen mass, which could be a gap in the process. We then weighed the yolk and albumen and analyzed the relationship between egg volume and shell area in relation to SD. Subsequently, 120 eggs were analyzed by Archimedes’ principle, including 60 with low density (between 1055 and 1072g/L) and 60 with high density (between 1079 and 1095g/L). Each egg was weighed, followed by separate weighing of the yolk, albumen, and eggshell (Maxx SK303; 3kg capacity; 0.1g precision), the latter after drying at 150°C for 2 hours to eliminate membrane weight. The relationship between the two methods was evaluated using Pearson’s correlation coefficient, while agreement and systematic bias were assessed using Bland–Altman analysis. The analyses were performed using Microsoft Excel 365. Bland–Altman analysis was performed using jamovi, version 2.7.30, available online (https://www.jamovi.org/).

As we observed good results with the Archimedes test, we performed this test on 3,374 eggs from four flocks (Supplementary Table 2), as described previously.

### Evaluation of the physical and geometric parameters and hatchability analysis

We worked with a total of 8,874 eggs sourced from five distinct flocks. At the outset, eggs from all flocks were included in the study. However, a traceability issue arose with one flock, limiting the use of its eggs exclusively to geometric measurements. As a result, geometric measurements were performed on eggs from all five flocks, whereas SD and water loss tests were conducted only in four flocks. Supplementary Table 2 summarizes the number of eggs used for each analysis.

All 8,874 tested eggs were analyzed geometrically, both by image analysis and manually. Egg length and width were measured using a digital caliper (Starfer®, 0.01mm). Based on length and width measurements, the area-volume ratio (AV) was calculated as the area over volume, according to Alkan et al. (2015); Besch et al. (1968); Karabulut (2021); Narushin (2001); Sauveur (1988); Wang et al. (2021), (Table 1). Moreover, shape index (SI) was calculated as width over length (Wang et al., 2021). Finally, egg weight was individually measured using a digital scale (Maxx SK303; 3kg capacity; 0.1g precision), and each egg was identified with a sequential number for tracking until hatching.

**Table 1:** Authors and their proposed equations for calculating egg area and volume.

Author(s) and Year of Publication	Proposed Formula for Area	Proposed Formula for Volume
Narushin et al., 2001	(3.142height²) * (1.057(height/width)^(−0.532)/100)	(0.49645* height*width²/11000
Wang et al., 2021	((widthheight²)^(1/3))²pi	((Pi/6)height*width²/1000
Sauveur, 1988	4.68*weight²/3	Not informed
Besch et al., 1968	4.76*weight^0.658	Not informed
Alkan et al., 2015	3.978*weight^0.7056	((0.6057-(0.018-width))*(height*width²)/100000
Karabulut, 2021	Not informed	((0.507height*width²)/1000

**Fig. 1:**
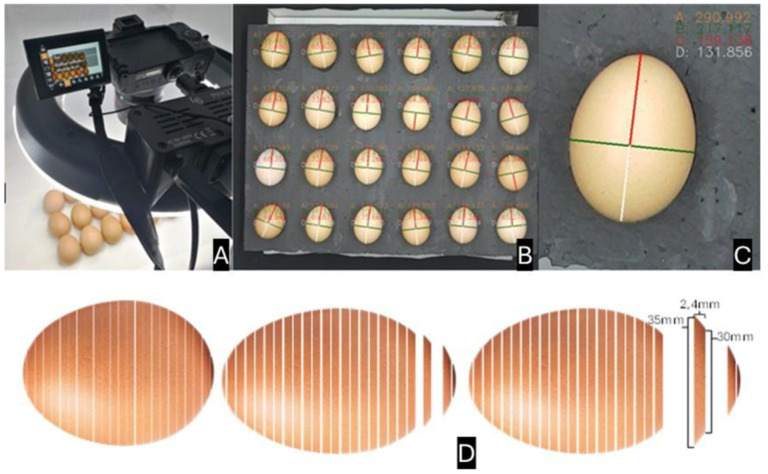
Illustration of the technique used for measuring the geometric characteristics of the eggs (brown eggs were used to illustrate the technique) A: Pilot experiment used to capture images in high resolution; B: 30-egg tray set up after image capturing, showing measurements in pixels regardless of exact egg orientation; C: Individual egg with measurements in pixel; D: sequence of partitioning of each egg for calculations of surface area and volume where red+white lines represent the height of the egg, while green light represent the largest width. Red line shows the length from the height: width intersection to the pointed end, while the white line shows the length from the height:width intersection to the blunt end of the egg.

The geometric characteristics of the eggs were digitally measured using a methodology developed in collaboration with the Image Processing Department of Faculty of Computer Science at the Federal University of Uberlândia (Tomé, 2024). A chamber and an algorithm were developed to measure egg width, height, area, volume, shape index (width divided by height), and area-to-volume ratio through the simultaneous imaging of 30 eggs at a time (Supplementary Figure 2). Briefly, the eggs passed through an imaging chamber, where images were captured. The algorithm then measured each egg and split it into one-pixel-resolution cross-sections. From this division, an egg-specific number of conical segments was generated and used to estimate egg volume and surface area The calculations involved identifying the contour of each egg and subsequently partitioning it to the pixel level. After partitioning, each egg was represented by several semi-cone segments. The total surface area and volume of each egg were then obtained by summing the area and volume of all semi-cone segments. Fig. 1 illustrates the conceptual basis of this technique.

After the measurements, the results obtained in the digital analyses were converted into millimeters. To assess the egg geometry calculations, we compared them with several methods proposed by different authors (Alkan et al. (2015); Besch et al. (1968); Karabulut (2021); Narushin (2001); Sauveur (1988); Wang et al. (2021)) (Table 1), and then performed correlation matrix analyses using jamovi, version 2.7.30, available online (https://www.jamovi.org/), comparing these results with those obtained from the digital evaluation. We considered p < 0.05 and R² values above 0.700 as indicative of a strong correlation.

A total of 8,874 eggs were individually identified in each tray using non-toxic wax crayons and/or pencils, and the incubation trays were marked with paper and adhesive tape. The eggs were incubated in Coopermaq multi-stage incubators, model 576, using trays holding 150 eggs, which were consistently positioned in the upper portion of the trolley during the first four incubation batches. For the final incubation flock, the trays were placed on a full trolley. The incubators were operated under constant settings, with a dry-bulb temperature of 99.7°F and a wet-bulb temperature of 85°F until transfer to the hatcher of the same brand (model KK192) at 19 days of incubation, where a dry-bulb temperature of 98.5°F and a wet-bulb temperature of 86°F were maintained.

At the time of transfer (456 hours of incubation), when embryos were moved from the setter to the hatcher, eggs from the first four incubation batches were individually weighed again using the same scale to calculate the moisture loss index. This index was obtained by dividing the difference between the initial weight and the transfer weight by the initial weight and expressing the result as a percentage. The identification of each incubation tray was transferred to the hatching basket to ensure traceability of each egg until embryo diagnosis. In each measurement, cracked or broken eggs identified as defective were excluded from the analyses, resulting in the total number of eggs reported in Supplementary Table 2.

At hatch, all chicks were counted and processed according to standard hatchery procedures, including sexing, grading, vaccination, and shipment. Unhatched eggs were separated for embryo diagnosis. The diagnostic evaluation classified unhatched eggs into the following categories: infertile, early mortality (0–7 days), mid mortality (8–14 days), late mortality (15–21 days), and contaminated.

Each geometric calculation, SD, egg weight, and weight loss (independent variables) were grouped into four percentiles (0.0–25.0%; >25.0–50.0%; >50.0–75.0%; and >75.0–100.0%) (Table 2). Using this grouping, pairwise chi-square analyses were performed to compare hatching and embryo diagnosis outcomes across quartiles of the independent variables. Statistical significance was set at p < 0.05, and the analyses were conducted using Microsoft Excel 365.

**Table 2:** Mean of the Results of specific density, weight loss, weight of the eggs, area, volume, area-to-volume ratio by image, and shape index in the eggs of each quartile analyzed.

Quartile	Q1 Mean (LL-UL)	Q2 Mean (LL-UL)	Q3 Mean (LL-UL)	Q4 Mean (LL-UL)
**Weight (grams)**	52.0a (38.1-53.9)	55.8b (54.0-56.7)	58.3c 56.8-59.8)	62.1d 59.9-76.2)
**Archimedes density**	1.065a (1030-1069)	1.072b (1070-1074)	1.077c (1075-1081)	1.083d (1082-1110)
**Weight loss (%)**	10.70%a (5.0-11.90)	12.70%b (11.91-13.10)	13.90%c (13.11-14.60)	16.40%d (14.61-30.80)
**Area (cm^2^)**	69.1a (56.10-73.30)	73.2b (73.31-76.50)	75.8c (76.51-79.80)	79.7d (79.81-101.50)
**Volume (cm^3^)**	50.5a (28.40-57.70)	55.8b (57.71-61.70)	60.2c (61.71-65.80)	65.3d (65.81-93.70)
**Area:Volume**	1,212a (1.08-1.21)	1,249b (1.22-1.24)	1,331c (1.25-1.27)	1,402d (1.28-2.00)
**Shape Index**	72.2%a (63.40-74.00)	74.7%b (74.01-75.80)	76.4%c (75.81-77.70)	79.0%d (77.71-87.40)

LL: Lower limit; UL upper limit. Q1: The eggs in the lower quartile. Q2: Eggs in the quartile between 25% and 50% of the results. Q3: Eggs in the quartile between 50% and 75% of the results. Q4: Eggs in the top 25% of the results. Different letters within a row indicate a statistically significant difference (p<0.05).

### Association between yolk minerals or metabolites and SD

Since we observed that SD had a greater influence on hatchability and that there is an association between low SD and infertility, we investigated yolk minerals and metabolites that could explain the relationship between yolk composition and SD and/or fertility. Our investigation was based on the fact that several minerals involved in fertilization are also present in the eggshell. Furthermore, little is known about the relationship between yolk metabolites and egg quality, and the use of non-targeted metabolomics may reveal potential associations, shedding light on this phenomenon and opening avenues for future studies. In total, 30 yolks were collected from each of the four flocks, including 15 from low-SD eggs (1055–1072g/L) and 15 from high-SD eggs (1079–1095g/L), totaling 120 yolk samples.

To evaluate whether any minerals in the yolk are associated with shell quality, as measured by SD, lyophilized yolk samples (0.2g) were treated with 3.0mL of HNO₃ (65%) and allowed to stand for 30 minutes. The digestion block was then gradually heated to 120°C for 60 minutes, until the complete release of brown NO₂ fumes. During this process, three aliquots of 500µL of H₂O₂ (30%) were added. At the end of the digestion, 1.0mL of HClO₄ (70%) was added, and the temperature was gradually increased to 150°C, at which it was maintained until complete digestion (3–4h). After cooling, the digests were transferred to 25mL volumetric flasks, and the final volume was made up with Milli-Q water. Following this procedure, a total of 103 yolks were of sufficient quality to be evaluated.

Elemental determinations were performed using an ICP-MS 7850 (Agilent Technologies, Japan), equipped with an SPS 4 autosampler and an ADS 2 autodilution system. Calibration curves were automatically generated by the ADS 2 system. Instrumental conditions followed the manufacturer’s application note, with measurements carried out in helium mode (He). Data acquisition and processing were performed using MassHunter software version 5.3 (Agilent Technologies), which also provided the detection limits (LOD) for each element. Calibration curves and LOD values were automatically calculated and validated by the software.

Mineral composition in biological matrices typically exhibits strong collinearity, heterogeneous scales, and the possibility of nonlinear and interaction effects on outcomes. Our dataset added the constraints of a small-n, p-moderate setting (although 120 samples were initially collected, 87 passed quality control after extraction and analysis; 11 minerals; complete cases, no imputation), where overfitting and unstable estimates are salient risks. To address these challenges and to triangulate evidence under different modeling assumptions, we implemented a complementary suite of methods spanning unsupervised, supervised linear, biologically structured, and nonparametric frameworks.

We first applied Principal Component Analysis (PCA) to standardized mineral concentrations to characterize the dominant covariance structure without conditioning on eggshell density. This unsupervised baseline reveals latent mineral axes (e.g., electrolyte balance versus trace/heavy-metal gradients) and provides a diagnostic of whether density differences align with major modes of variation through post hoc regressions of density on PC scores. To evaluate linear associations in the presence of strong multicollinearity, we then used Partial Least Squares (PLS) and lasso regression. PLS extracts components that maximize covariance with density and, through cross-validation, determines the effective dimensionality; Variable Importance in Projection (VIP) scores rank minerals by their contribution to the predictive subspace. Lasso complements PLS by performing shrinkage and variable selection directly in the original feature space. Because physiological processes often manifest more strongly in ratios than in absolute concentrations, we constructed domain-informed predictors (e.g., Na:K, Ca:Fe, Zn:Cu) capturing ionic balance, structural versus redox functions, and toxic load relative to essential minerals. These predictors were evaluated in univariate and multivariable linear models (using safe ratio definitions to avoid division artifacts), providing explicit tests of biologically motivated hypotheses that might be obscured by collinearity. To assess nonlinear relationships and potential interactions without imposing a parametric form, we fit Random Forest (RF) regression with permutation importance and out-of-bag (OOB) validation. RF asks whether predictive structure exists beyond linear/additive modeling and highlights variables or ratios that matter most when arbitrary decision boundaries are allowed. Design choices prioritized robustness and interpretability: data were expressed as mg/kg; repeated measures per analyte were collapsed using per-sample medians to limit batch/outlier influence; predictors were standardized for supervised models; and complete-case analyses were conducted to avoid fabricating signal in a small sample. PLS and lasso used K-fold cross-validation, whereas RF relied on OOB performance for unbiased generalization estimates. We report VIP values (PLS), non-zero coefficients (lasso), permutation importance, and partial-dependence profiles (RF), along with classical summaries for ratio models, enabling triangulation across modeling assumptions. Agreement across these independent frameworks strengthens inferences about mineral–density relationships; disagreement suggests that any effects are small, unstable, or nonlinear relative to the available sample size.

To evaluate whether any yolk metabolites are associated with shell quality, as measured by SD, eggs classified as high (1079–1095g/L) or low (1055–1072g/L) SD were weighed and lyophilized to standardize sample quantity. For each sample, 100mg of lyophilized yolk was mixed with 1,000µL of spectroscopic-grade methanol, followed by vortex homogenization and centrifugation for 15 minutes at 13,000g. The supernatant was then transferred to a new microcentrifuge tube and subjected to vacuum concentration for approximately 18 hours until completely dry. The resulting material was resuspended in 400µL of methanol and filtered through a 0.22µm filter.

Metabolite analysis was performed using HPLC-MS equipped with a high-performance liquid chromatograph (Agilent Infinity 1260) coupled to a high-resolution Q-TOF mass spectrometer (Agilent® model 6520B) with an electrospray ionization (ESI) source. Separation was achieved using an Agilent Poroshell 120 column (2.1 × 50mm, 2.7µm). The mobile phase consisted of formic acid-acidified water (0.1% v/v) (A) and methanol (B), with the following gradient: 10% B (0min), 98% B (0-10min), and 98% B (10-17min). The ionization parameters were a nebulizer pressure of 20psi, drying gas flow of 8L/min at 220°C, and a capillary voltage of 4.5kV. The injection volume was 4µL.

The raw data were processed using MassHunter Qualitative software v. 10.0. The Molecular Feature Extraction (MFE) tool was used to extract mass spectra and convert them to the “.cef” file format. Filtering and analysis of the extracted molecular compounds were performed using Agilent Mass Profiler Professional (MPP) software v. B.13.1.1. The applied filters included a minimum absolute abundance of 5,000 counts, with all charge states allowed. The analysis parameters were retention time tolerance (0.15min) and mass window (15ppm + 2 mDa). Relevant molecular compounds were defined as those present in at least 75% of samples within one group.

Metabolites were identified using the METLIN 2019 database (included in MPP), which contains entries derived from plants and fertilizers. Therefore, isomer evaluation was required. For isomer analysis, data from the mass spectrometry files in MassHunter software were used to obtain the exact mass of the metabolites under study. Subsequently, the Human Metabolome Database (HMDB; available at https://www.hmdb.ca/spectra/ms/search) and the Metabolomics Workbench (available at https://www.metabolomicsworkbench.org/databases/metabolitedatabase.php) databases were used.

Agilent Mass Profiler Professional (MPP) v. B.13.1.1 was used for statistical analysis. The data were log₂-transformed and analyzed using unpaired t-tests. Metabolites with statistical significance set at p < 0.05 and a fold change (FC) equal to or greater than 2.00 were considered differentially abundant. Once the validity of the metabolites between the two groups was confirmed (p < 0.05 and FC > 2), heatmaps were constructed for enhanced visualization. Subsequently, simple regression analyses were performed to evaluate sensitivity and area under the curve (AUC) values using GraphPad Prism software (version 10.0).

## Results

### Preliminary results

The comparison between the Archimedes method and the salt-solution method for measuring egg SD demonstrated that the two methods were moderately correlated (r = 0.466; p < 0.05). However, Bland–Altman analysis revealed a clear and statistically significant systematic bias of −5.07 units (−5.31 to −4.82, 95%CI), which exceeds the regular grading interval of the saline method most commonly applied in commercial hatcheries. This indicates that one method consistently produces lower values than the other, and that the limits of agreement are wide, reflecting substantial variability between methods. Based on these differences, we adopted the Archimedes method for this experiment. No differences were observed in yolk or albumen weight, nor in egg volume or surface area, when comparing high-density and low-density eggs (Table 3).

**Table 3:** Egg components according to the density category of the eggs by the Archimedes’ principle.

	High Density	Low Density	P-value
**Average Density (g/L)**	1.086	1,067	0.0001
**Egg weight (g)**	56.1	56.3	0.870
**Volume (cm^3^)**	54.2	54.0	0.922
**Area (cm^2^)**	72.5	72.5	0.979
**Shell weight (g)**	6.1 a	5.3 b	0.0001
**Shell weight/volume (g/cm^3^)**	105.2	97.4	0.073
**Shell weight/area (g/cm^2^)**	84.2 a	74.2 b	0.0001
**Yolk weight (g)**	16.0	16.2	0.571
**Albumen weight (g)**	34.1	34.8	0.319
**% Yolk over egg weight**	28.5%	28.8%	0.519
**% Shell over egg weight**	10.7% a	9.5% b	0.0001

Different letters within a row indicate a statistically significant difference.

### The digital analysis is adequate to evaluate the shape of the eggs

We observed a high correlation between the manual analysis of the egg shape according to the methodology of several authors and the digital analysis, except for the egg area according to Besch et al. (1968) (Supplementary Table 3 and 4).

### The hatchability is mainly influenced by SD, and low SD has a high correlation with infertility and early embryonic mortality

The results of SD, weight loss, egg weight, area, area-volume ratio, volume and shape index and the number of eggs in each quartile are described in Table 2 and Supplementary Table 5.

There were no differences in hatchability among the quartiles of the eggs when comparing weight, area, and area-to-volume ratio. Regarding weight loss, the highest hatchability rates were observed in eggs from Q2 and Q3, corresponding to 11.91-14.60% weight loss, with hatchability values of 88.70% and 89.23%, respectively (Table 2, Table 4). However, hatchability in Q2 was also similar to that observed in Q1 and Q4. The shape index showed better hatchability (89.00%) in eggs from Q3, corresponding to 75.81-77.70 SI shape index, but it was similar to Q2 (86.00%) and Q1 (86.30%). Eggs in Q3 and Q4, corresponding 1075 – 1110g/L of SD, showed higher hatchability rates of 89.10% and 88.70%, respectively, compared with eggs in Q1 and Q2, which showed hatchability rates of 83.50% and 86.40%, respectively.

**Table 4:** Hatchability (%) based on the distribution (quartile) of egg`s weight, specific density, weight loss, area, volume, area: volume ratio and shape index.

Variable	Q1	Q2	Q3	Q4	
**Weight (grams)**	86.1%	87.6%	87.1%	85.8%	
**Specific density (g/L)**	83.5%b	86.4%ab	89.1%a	88.7%a	
**Weight loss (%)**	85.7%b	88.7%ab	89.2%a	84.6%b	
**Area (cm^2^)**	86.2%	86.9%	87.2%	86.2%	
**Volume (cm^3^)**	87.0%	86.6%	87.2%	85.8%	
**Area:Volume Image**	86.3%	86.5%	86.4%	87.3%	
**Shape Index**	86.0%ab	86.3%ab	89.0%a	85.3%b	

Q1: 1030-1069g/L to specific density, 5.0-11.90% to weigh loss, 63.40-74.00 to shape index; Q2: 1070-1074g/L to SD, 11.91-13.10% to weigh loss, 74.01-75.80 to shape index; Q3: 1075-1081g/L to SD, 13.11-14.60% to weigh loss, 75.81-77.70 to shape index; Q4: 1082-1110g/L to SD, 14.61-30.80% to weigh loss, 77.71-87.40 to shape index. Different letters within a row indicate a statistically significant difference (p<0.05).

There were no differences in infertility among egg quartiles when eggs were classified according to weight, weight loss, volume, area-to-volume ratio, or shape index. However, eggs in Q1 and Q2, corresponding to 1030–1074g/L of specific density measured using Archimedes’ principle, showed lower infertility rates than those in Q3 and Q4 (1.24% and 1.11% vs. 2.43% and 2.40%, respectively; Table 2, Table 5).

**Table 5:** Infertility (%) based on the distribution (quartile) of egg weight, specific density, weight loss, area, volume, area-to-volume ratio by image, and shape index.

Variable	Q1	Q2	Q3	Q4
**Weight (grams)**	2.17%	1.27%	1.59%	2.04%
**Specific density (g/L)**	2.43%a	2.40%a	1.24%b	1.11%b
**Weight loss (%)**	1.88%	1.45%	1.80%	2.10%
**Area (cm^2^)**	2.39%a	1.61%ab	1.06%b	1.98%ab
**Volume (cm^3^)**	2.06%	1.37%	1.27%	2.32%
**Area:Volume Image**	2.22%	1.58%	1.27%	2.04%
**Shape Index**	1.93%	1.48%	1.39%	2.29%

Q1: 1030-1069g/L to specific density, 56.10-73.30 cm^2^ to area; Q2: 1070-1074g/L to SD, 73.31-76.50 cm^2^ to area; Q3: 1075-1081g/L to SD, 76.51-79.80 cm^2^ to area; Q4: 1082-1110g/L to SD, 79.81-101 cm^2^ to area. Different letters within a row indicate a statistically significant difference (p<0.05).

No significant differences in embryonic mortality were observed among egg quartiles when comparing early, mid, and late deaths with respect to weight, area, volume, or area-to-volume ratio, as determined by digital analysis. However, for early mortality, eggs in Q1 and Q2 of Archimedes density (Table 2) showed higher mortality (Q1: 5.97% and Q2: 5.22%) compared with those in Q3 (4.02%), although values were similar to those in Q4 (4.35%) (Table 2, Table 6). The Q4 of shape index also exhibited the highest early mortality (5.57) compared to Q3 (3.53%), but values were similar to those observed in Q1 (5.03%) and Q2 (4.98%). In contrast, for mid mortality, eggs in Q1 showed lower mortality (0.00%) associated with weight loss compared with the other quartiles. For late mortality, weight loss was associated with higher mortality in eggs from Q1 (6.91%) and Q4 (5.02%) compared with those from the middle quartiles (Q2: 3.23% and Q3: 3.48%).

**Table 6:** Embryonic mortality (%) based on the distribution (quartile) of specific density, weight loss, egg weight, area, volume, area-to-volume ratio by image, and shape index.

Embryonic Mortality	Quartile	Weight (grams)	Archimedes density	Weight loss (%)	Area (cm^2^)	Volume (cm^3^)	Area:Volume Image	Shape Index
**Early dead**	Q1	4.45%	5.97%a	4.64	4.66%	4.59%	5.17%	5.03%ab
	Q2	5.17%	5.22%a	4.89	4.71%	4.86%	4.74%	4.98%ab
	Q3	5.24%	4.02%b	4.38	4.75%	4.86%	4.98%	3.53%b
	Q4	4.49%	4.35%ab	5.65	5.22%	5.06%	4.40%	5.74%a
**Middle dead**	Q1	0.31%	0.22%	0.00%a	0.34%	0.34%	0.42%	0.75%
	Q2	0.32%	0.73%	0.78%b	0.32%	0.32%	0.42%	0.42%
	Q3	0.34%	0.21%	0.34 b	0.42%	0.53%	0.42%	0.21%
	Q4	0.61%	0.45%	0.55b	0.52%	0.42%	0.32%	0.21%
**Late dead**	Q1	5.17%	5.75%	6.91a	4.55%	4.01%	4.44%	3.85%
	Q2	4.22%	4.28%	3.23b	5.25%	5.71%	5.06%	5.41%
	Q3	4.21%	4.44%	3.48b	4.65%	4.54%	5.51%	4.38%
	Q4	5.41%	4.79%	5.20a	4.59%	4.75%	4.08%	5.42%

Q1: 1030-1069g/L to specific density, 5.0-11.90% to weigh loss, 63.40-74.00 to shape index; Q2: 1070-1074g/L to SD, 11.91-13.10% to weigh loss, 74.01-75.80 to shape index; Q3: 1075-1081g/L to SD, 13.11-14.60% to weigh loss, 75.81-77.70 to shape index; Q4: 1082-1110g/L to SD, 14.61-30.80% to weigh loss, 77.71-87.40 to shape index. Different letters within a column indicate a statistically significant difference (p<0.05).

No significant differences in contamination were observed among egg quartiles when comparing weight, area, volume, or area-to-volume ratio. However, eggs in Q1 of Archimedes density exhibited higher contamination (2.10%) than those in the other quartiles (Q2: 0.94%, Q3: 1.03%, Q4: 0.56%) (Table 7). The highest contamination associated with weight loss was observed in eggs from the Q4 (1.88%), with values similar to those in Q2 (0.79%). Shape index indicated lower contamination in Q4 (1.04%) compared to Q1 (2.46%), although values of Q4 were similar to those observed in Q2 (1.38%) and Q3 (1.50%) (Table 2, Table 7).

**Table 7:** Egg contamination (%) based the distribution (quartile) of egg weight, specific density, weight loss, area, volume, area-to-volume ratio by image, and shape index.

Quartile	Q1	Q2	Q3	Q4
**Weight (grams)**	1.76%	1.48%	1.48%	1.63%
**Specific density (g/L)**	2.10%a	0.94%b	1.03%ab	0.56%b
Weight loss (%)	0.89%b	1.00%ab	0.79%b	1.88%a
**Area (cm^2^)**	1.82%	1.18%	1.90%	1.46%
**Volume (cm^3^)**	1.95%	1.16%	1.58%	1.69%
**Area:Volume Image**	1.48%	1.69%	1.38%	1.82%
**Shape Index**	2.46%a	1.38%ab	1.50%ab	1.04%b

Q1: 1030-1069g/L to specific density, 5.0-11.90% to weigh loss, 63.40-74.00 to shape index; Q2: 1070-1074g/L to SD, 11.91-13.10% to weigh loss, 74.01-75.80 to shape index; Q3: 1075-1081g/L to SD, 13.11-14.60% to weigh loss, 75.81-77.70 to shape index; Q4: 1082-1110g/L to SD, 14.61-30.80% to weigh loss, 77.71-87.40 to shape index. Different letters within a row indicate a statistically significant difference (p<0.05).

### Even weak Na:K ratio in Yolk is associated with SD

**Fig. 2:**
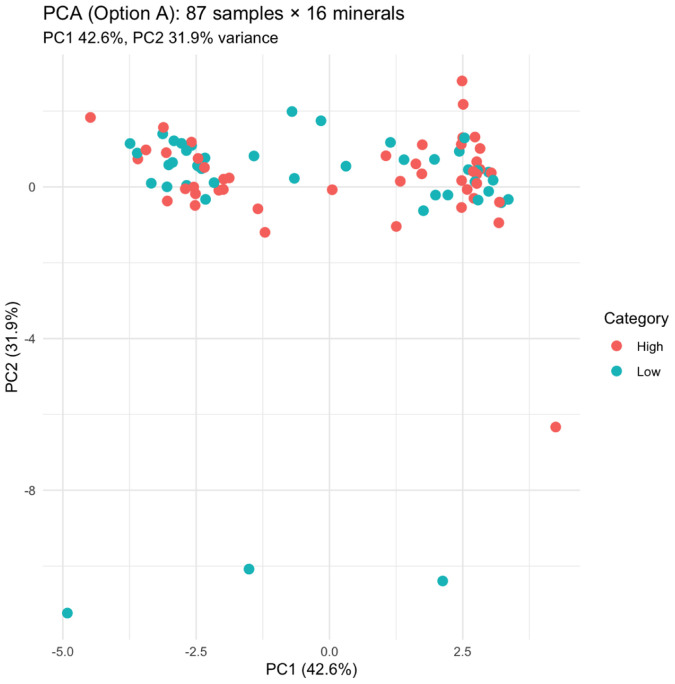
Principal Component Analysis of Mineral Profiles in eggs with high and low specific density. Scores plot of PC1 *vs.* PC2 from a PCA performed on the standardized Option A matrix (87 samples × 16 minerals, mg/kg; complete cases; no imputation). Points represent individual samples, colored by Category (high, low). Axis labels report the percent of variance explained by each PC. This plot summarizes the dominant covariance structure among minerals without using density in the fit.

Mineral concentrations exhibited a strong covariance structure (PC1: 42.6%; PC2: 31.9%), but high- and low-density groups showed extensive overlap in PCA space, and density was not associated with either principal axis (PC1: β = 0.48, p=0.30; PC2: β = 0.58, p=0.27) (Fig. 2). No individual mineral differed significantly between density groups in univariate tests (Table 8). Supervised models consistently supported a weak relationship: PLS explained little variance under cross-validation (RMSEP lowest at 1–2 components, ≈ 11, increasing with higher dimensionality) with low VIP scores (Fe = 0.71; Cr = 0.63), and lasso retained no stable predictors at the 1-SE penalty. Random Forest regression showed no improvement over the null model (OOB R² ≈ −0.02; RMSE ≈ 11.18), and adding interaction terms did not improve accuracy (OOB R² ≈ −0.03).

**Table 8:** Mean levels of minerals (mg/kg) in the egg yolk of low and high density.

Analyte	High Density	Low Density
**Ca**	2,925	2,878
**Na**	2,127	2,064
**K**	1,696	1,762
**Mg**	248	252
**Fe**	152	136
**Zn**	24.63	29.06
**Co**	0.01	0.04
**Cr**	0.87	0.92
**Mn**	0.75	0.72
**Se**	0.18	0.26
**Cu**	0.00	0.00

**Fig. 3:**
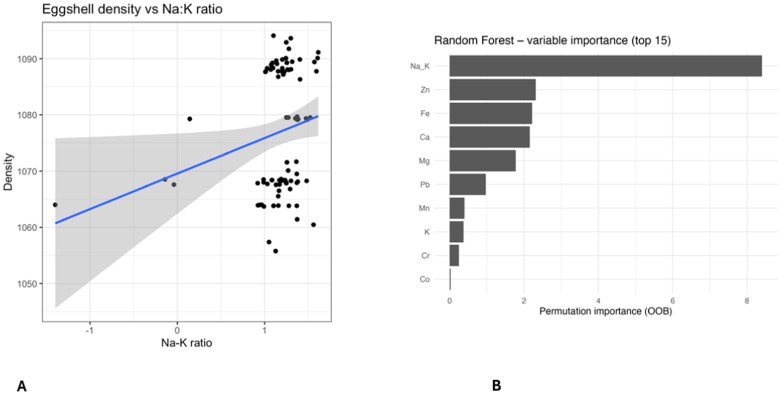
Univariate regression between Na:K ratio and eggshell quality, and Random Forest regression predictive value of biological mineral ratios and individual minerals.

Biologically informed ratios (Na:K, Ca:Fe, Zn:Cu) showed only modest linear trends with small effect sizes. Although the Na:K ratio showed a significant but weak univariate association with density (β = 6.32, p = 0.035; R² = 0.051), this effect was not supported in a multivariable model (β = 6.85, p = 0.134; R² = 0.052), and Ca:Fe and Zn:Cu were not significant. Permutation importance consistently ranked Na:K as the most influential feature across models (Fig. 3B), yet partial dependence profiles revealed only a shallow gradient with a subtle inflection around Na:K ≈ 1, without strong thresholds. Taken together, these convergent results indicate that mineral composition alone has limited explanatory power for eggshell density, and that structural or organic components likely play a more dominant role in determining shell integrity.

### Melatonin and long-chain fatty acids in the yolk are associated with SD

In total, we identified 310 metabolites; however, only two passed the 75% abundance filter with p < 0.05 and a fold change > 2. Initial metabolite identification using the METLIN library proposed the compounds olomoucine (m/z 299.1616) and eudesmin (m/z 409.1606). After verification, these compounds were discarded, as they are of synthetic origin and a plant-derived metabolite, respectively. Subsequent database searches based on the m/z values led to new candidate identifications, such as melatonin glucuronide and dihydroxytetradecanoic acid, the latter being one of the isomers suggested with an acceptable mass error (Supplementary Table 6).

**Fig. 4:**
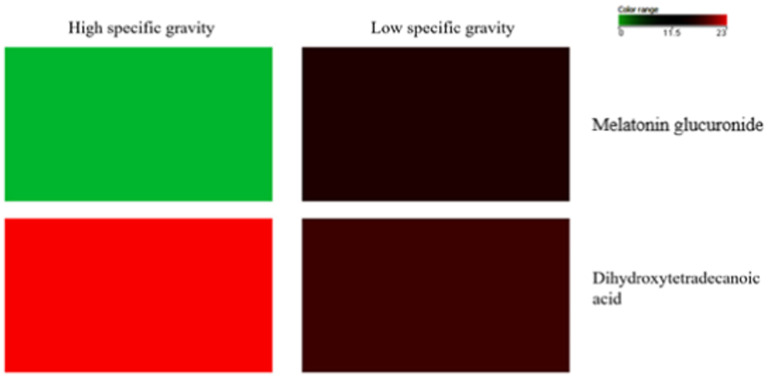
Heatmap of the Melatonin glucuronide and Dihydroxytetradecanoic in the groups of high and low specif density Acid.

Melatonin glucuronide was more abundant in eggs with low SD (p < 0.05) (Fig. 4). Dihydroxytetradecanoic acid was more abundant in eggs with high SD (p < 0.05) (Fig. 4). Although differences in mean abundance were observed, logistic regression showed low AUC values (0.53 for dihydroxytetradecanoic acid and 0.58 for melatonin glucuronide), both close to chance level (AUC = 0.50), indicating that these metabolites alone have very weak discriminative ability and cannot reliably distinguish between high- and low-density eggs.

## Discussion

In our preliminary studies, we observed moderate correlation between the saline solution method and Archimedes’ method. In addition, the Bland–Altman analysis showed a significant mean difference of 5.07g/L (−5.31 to −4.32, 95%CI), indicating that the results obtained using the saline solution method may differ from those obtained using Archimedes’ method by approximately 5.07g/L. This difference corresponds to the interval commonly used in the saline solution scale. Therefore, an egg classified into a specific gravity category by one method may be allocated to an adjacent category when assessed using the other method, due to methodological differences.

The saline methodology, in routine practice, is more prone to human error, as the results are influenced by factors such as the densimeter, the observer's eye height, and the speed of the process. Additionally, transferring eggs between solutions alters the solution's density, requiring constant recalibration. There were also uncertainties regarding whether the analysis was influenced by the weight of the albumen or yolk, rather than the eggshell weight. In contrast, our results showed that the yolk, albumen, egg surface area, and volume do not interfere with the measurements obtained using Archimedes' principle (Table 3), suggesting that this test is more reliable. Therefore, for this study, we chose to use the Archimedes' principle measurement.

Another point in our work was related to the digital analysis of the egg's geometric characteristics. We observed that the digital analysis was correlated with the manual methodology of all the authors studied (Supplementary Tables 3 and 4). Therefore, considering that digital analysis is fast and has a lower possibility of human error, digital analysis is suitable for studies on egg geometric characteristics.

Higher hatchability was observed in eggs with intermediate weight loss (25-75% - 1.95-14.60%) (Table 4), and this improved hatchability is associated with the lower late embryonic mortality found in eggs with moderate weight loss (Table 6). This is probably due to different reasons, since eggs with low weight loss tend to have a smaller air cell, which can make internal pipping more difficult and hinder the transition from chorioallantoic respiration to pulmonary respiration (Molenaar et al., 2010). On the other hand, high weight loss percentages result in a very large air cell, probably formed too early in development, leading to malpositions later in life, which compromises hatchability (Molenaar et al., 2010). We also observed a lower middle embryonic mortality in eggs with weight loss lower than 11.90% (Table 6), but these results did not affect hatchability (Table 4). The biological basis of this finding remains unclear. However, since the middle embryonic mortality was low and no chicken embryos died in this group, this result should be interpreted cautiously.

Shape index is essential for preventing the incorrect placement of hatching eggs on setter trays (Icken et al., 2006). Our results showed that the shape index was associated with better hatchability in eggs from the middle 50% to 75% (75.81-77.7), but it was similar to the middle 25%−50% and bottom 25% (<75.81) (Table 4). This point varies significantly among different studies. While one study shows that intermediate values of SI are associated with higher hatchability (Segura et al., 2024), others have found that rounder eggs (higher shape index) tend to hatch better (Alasahan and Copur, 2016; Narushin and Romanov, 2002). Narushin and Romanov (2002) report that, although extreme egg shapes are detrimental, embryonic development is more favorable in eggs with a breadth-to-length ratio slightly below the flock average, indicating an advantage for moderately elongated eggs compared with excessively rounded ones. Although the available literature is still insufficient to fully support our results or explain the discrepancies among studies, both narrow and markedly round egg shapes could restrict embryo rotation inside the egg (Narushin and Romanov, 2002). In the case of spherical eggs, it may be difficult to identify the blunt end, which can lead to their placement in the incubator with the air cell facing downward, thereby reducing the likelihood of hatching. In elongated eggs, on the other hand, the embryo may become incorrectly positioned when shifting from a horizontal to a vertical orientation, leading to death in the initial stages of development (Biesiada-Drzazga, 2020). Interestingly, higher hatchability was associated with lower early embryonic mortality in eggs within the Q3 (75.81-77.70) of SI compared to those in Q4 (≥ 77.71). Although our results are consistent with previous studies reporting an association between increased early mortality and eggs with higher SI (Alasahan and Copur, 2016; Segura et al., 2024), the mechanisms responsible for this pattern have not yet been identified. Additional studies are required to clarify the biological basis of this relationship.

A robust result was observed for SD, with higher hatchability in eggs with greater SD (Table 4) (upper 50% quartile, ≥ 1075). A positive association between SD and hatchability is explored in the scientific literature, where higher SD is associated with higher hatchability (Bennett, 1992; Roque and Soares, 1994; Wolc et al., 2010) due to lower embryonic intermediate and late mortality (Roque and Soares, 1994). However, our results showed that the higher hatchability associated with SD can be explained by differences in infertility and early mortality (Tables 4, 5, and 6), as these two parameters were lower in eggs within the upper 50% quartile (≥ 1075g/L) of SD. We hypothesize that part of the early embryonic mortality may be explained by contamination, as eggs in Q1 (1030-1069g/L) SD group showed higher contamination levels (Table 7). Our experiment showed that eggs with lower SD have thinner shells, which is in agreement with (Fathi et al., 2022). A lower eggshell density can favor the entry of pathogens, increasing the risk of contamination in the early incubation stages (Neto et al., 2024).

Regarding the association between fertility and SD, our findings are consistent with previous studies reporting the same pattern (Roque and Soares, 1994; Segura et al., 2024). In the present study, we aimed to investigate candidate biological factors associated with both SD and fertility. Then, we studied the metabolites and minerals in the yolk, hypothesizing that a general physiological process in hens could impact both fertility and SD, even though yolk deposition in the ovary and shell formation in the uterus are distinct processes occurring at different times.

We did not find significant differences in individual yolk minerals between eggs with high and low SD (Fig. 2; Table 8). However, although weak, we observed an association between the yolk Na:K ratio and SD. Nevertheless, SD and fertility are complex traits that are unlikely to be explained by a single mineral ratio. Other yolk components not evaluated in the present study, such as lipoproteins, lipid classes, proteins, or interactions among minerals, metabolites, and macromolecules, may also contribute to differences in SD and fertility. Therefore, the Na:K ratio should be interpreted as a potential candidate associated with SD, but not as an isolated explanatory factor for egg specific density or fertility.

This finding is biologically relevant because sodium and potassium help maintain membrane potential and regulate the fluxes of Ca²⁺ and Zn²⁺ involved in fertilization (Gałęska et al., 2022). Evidence indicates that Na⁺ and K⁺ are involved in reproductive and eggshell-forming processes. In fertilization, the ionic composition of sperm plasma is associated with fertilization capacity, and changes in Na⁺ and K⁺ permeability occur during oocyte activation, together with the Ca²⁺ oscillations required for the initiation of embryonic development (Anifandis et al., 2019; Yeste et al., 2019). These ions are also relevant to follicular physiology, as sodium contributes to follicle viability and estrogen synthesis, while changes in potassium levels have been associated with follicular maturation in mammals (Hassan et al., 2018). In the shell gland, Na⁺ and K⁺ help maintain the electrochemical gradients required for Ca²⁺ and HCO₃⁻ transport, supporting calcium carbonate deposition and eggshell mineralization (Arad et al., 1989; Jonchère et al., 2012). Thus, although Na⁺ and K⁺ are not structural components of the shell matrix, they contribute to the ionic and physiological conditions involved in fertilization, follicular function, and eggshell formation. These mechanisms support the biological relevance of investigating the Na:K ratio in relation to SD and fertility. Although the association observed in the present study was weak, it should not be disregarded, as it may reflect the participation of the Na ratio in broader biological mechanisms involving other substances or events not evaluated here. Therefore, rather than being interpreted as an isolated explanatory factor, the Na:K ratio should be considered a novel candidate marker that may help guide future studies on the biological mechanisms linking SD and fertility.

Non-target metabolomics showed that melatonin glucuronide was more abundant in eggs with low SD and dihydroxytetradecanoic acid were more abundant in eggs with high SD (Fig. 4). Although t-test showed significant differences in the melatonin glucuronide, and dihydroxytetradecanoic acid between groups, the low AUC values indicate that these variables have limited discriminatory ability when evaluated individually. Rather than representing strong standalone biomarkers, these variables should be considered candidate markers associated with SD and fertility-related outcomes, requiring further investigation in combination with other biological factors.

Melatonin glucuronide is one of the secondary metabolites resulting from the processing of melatonin. As there is an association between eggs with low SD and low fertility, and a higher melatonin glucuronide level in eggs with lower SD, this suggests that altered melatonin metabolism may be linked to reproductive performance in birds, affecting both egg quality and infertility.

Regarding SD, melatonin supplementation in laying hens leads to increased bone strength at the expense of reduced eggshell thickness (Çalişlar et al., 2018). Thus, the elevated levels of melatonin glucuronide in the yolk of low SD eggs are consistent with altered calcium allocation, prioritizing skeletal health over the formation of thicker eggshells (Çalişlar et al., 2018).

Melatonin influences reproductive timing, and although it is important for reproduction, high levels negatively modulate the hypothalamic pituitary gonadal axis by inhibiting GnRH release. Changes in its metabolites could reflect disruptions in endocrine regulation that impair follicle development and oocyte maturation (ViviD and Bentley, 2018). Different melatonin receptors are present in the ovaries of hens, but there are contradictory results between authors regarding reproduction (ViviD and Bentley, 2018). To date, the specific mechanisms through which melatonin influences fertilization in birds remain unknown. One study shows that melatonin enhances the proliferative capacity of granulosa cells and suppresses apoptosis through receptor-mediated activation of the mTOR signaling pathway, which may improve fertility (Hao et al., 2020). Nevertheless, evidence from mammalian models indicates that melatonin can enhance fertilization efficiency, potentially through its well-established antioxidant effects (Dai et al., 2017; Gutiérrez-Añez et al., 2021; Tan et al., 2016; Veiga et al., 2024). The exact mechanism of how and to what extent melatonin affects reproduction and fertilization is not fully understood and needs further exploration (ViviD and Bentley, 2018). Together, these findings support the hypothesis that balanced melatonin metabolism is relevant to reproductive outcomes and egg quality. Our findings shed light on other studies to better understand how melatonin influences egg quality and specially in infertility.

We found several isomers of dihydroxytetradecanoic acid (Supplementary Table 6) that were more abundant in eggs with high SD (Fig. 4). However, no relationship was found between any isomer and the study. In humans, the database cites that 3,9-dihydroxytetradecanoic acid is directly involved in an organism’s growth, development, and reproduction (HMDB, 2022). Although we didn’t find studies that support our results, the isomers found are fatty acids of type FA 14:0;O2, with 14 carbon atoms, only single bonds, and two hydroxyl groups. Long-chain fatty acids (LCFAs) are important components of the yolk and can influence reproductive performance, egg quality, membrane fluidity, oxidative balance, and embryonic metabolism. In breeder hens, dietary enrichment with omega-3 long-chain fatty acids modifies the biochemical profile of hatching eggs and may support early embryonic viability (Thanabalan and Kiarie, 2021). Moreover, exposure to LCFAs, such as oleic acid, alters the expression of genes involved in lipid uptake, β-oxidation, and metabolic regulation in embryonic tissues (Zhang et al., 2023). In our study, all birds received the same diet, indicating that the higher abundance of dihydroxytetradecanoic acid isomers in high-SD eggs was not diet-derived. Although the underlying mechanism remains unclear, these findings suggest that these fatty acid derivatives may be associated with yolk composition and egg quality, warranting further investigation.

It is important to note that we classified eggs based on their SD without considering fertility status, and the majority of them were fertile. This represents a limitation of our study, and future research using infertile eggs could provide more accurate insights into the relationships we have explored.

## Conclusions

With a large number of samples, our work confirms that, although extreme values of weight loss or shape index can negatively affect hatchability, SD was the factor most strongly associated with hatchability outcomes in the present study. Beside the number of samples, robustness of our work is supported by the use of improved and advanced methodologies, such as digitally derived geometric characteristics and specific density measured using Archimedes’ principle. Eggs with lower SD values exhibited higher rates of infertility and early embryonic mortality. The yolk Na:K ratio showed a significant but weak association with SD. Eggs with high SD exhibited lower levels of melatonin glucuronide and higher levels of dihydroxytetradecanoic acid in the yolk. However, these substances alone do not fully explain low SD or reduced fertility. Therefore, they should be interpreted as potential biomarkers that may contribute, together with other biological events or substances not investigated in the present study, to a better understanding of the relationship between SD and fertility.

## Author contribution statement

**Thomas A.C. Calil** contributed to the conception of the study, sample collection, data analysis, interpretation of the results, and critical discussion of the manuscript. **Bruno S. Vieira** contributed to statistical analysis, interpretation of the results, and critical discussion of the manuscript. **Simone Sommerfeld** contributed to sample collection, metabolomic analyses, interpretation of the results, and critical discussion of the manuscript. **Arlene B. dos S. Nossol** contributed to sample collection, mineral analyses, interpretation of the results, and critical discussion of the manuscript. **Tiara da C. Silva** contributed to sample collection, mineral analyses, interpretation of the results, and critical discussion of the manuscript. **Douglas Q. Santos** contributed to mineral analyses, interpretation of the results, and critical discussion of the manuscript. **Ana Clara F. Silva** contributed to sample collection, egg quality analyses, interpretation of the results, and critical discussion of the manuscript. **Mário M. Martins** contributed to metabolomic and mineral analyses, interpretation of the results, and critical discussion of the manuscript. **Victor A. de S. Penha** contributed to statistical analyses, data interpretation, and critical discussion of the manuscript. **Mauricio C. Escarpinati** contributed to the development and application of computational analyses, data interpretation, and critical discussion of the manuscript. **Hebréia O. Almeida-Souza** contributed to metabolomic analyses, interpretation of the results, and critical discussion of the manuscript. **Marcelo E. Beletti** contributed to metabolomic analyses, functional interpretation of the data, and critical discussion of the manuscript. **Anna Luiza F.D. Strack** contributed to laboratory analyses, interpretation of the results, manuscript drafting, and critical discussion of the manuscript. **Belchiolina B. Fonseca** conceived and supervised the study, contributed to the investigation, data analysis, interpretation of the results, manuscript preparation/edition, and critical discussion of the manuscript.All authors contributed intellectually to the study, critically reviewed the manuscript, approved the final version, and agree to be accountable for their respective contributions.

## Disclosures

The authors declare that they have no known competing financial interests or personal relationships that could have appeared to influence the work reported in this paper.

## Appendix Supplementary materials

Image, application 1

## Footnotes

Supplementary material associated with this article can be found, in the online version, at doi:10.1016/j.psj.2026.107356.
