# Supplementary material for: Yolk metabolomics reveals candidate compounds associated with egg specific density and hatchability in white layer breeder hens
Source: Poult Sci. 2026 Jun 29;105(10):107356. doi: 10.1016/j.psj.2026.107356 (PMC13380467; doi:10.1016/j.psj.2026.107356)
Supplement: Supplementary file 1 [file mmc1.docx]

**SUPPLEMENTARY DATA**

**Supplementary Table 1**. Nutritional levels adopted for the flocks of layer hens tested in the experiment

| **Nutrient** | **Unit** | **Levels** | **Nutrient** | **Unit** | **Levels** |
| --- | --- | --- | --- | --- | --- |
| **ME Birds** | Kcal/kg | 2,755 | Digestible Tryptophan | % | 0 |
| **Crude Protein** | % | 16 | Digestible Arginine | % | 0.922 |
| **Ether Extract** | % | 3 | Digestible Valine | % | 0.691 |
| **Crude Fiber** | % | 3.17 | Digestible Leucine | % | 1.304 |
| **Mineral Matter** | % | 13.5 | Digestible Isoleucine | % | 0.603 |
| **Total Calcium** | % | 4.24 | Linoleic Acid C18:2, n-6 | % | 1.469 |
| **Total Phosphorus** | % | 0.44 | Xanthophylls | mg/kg | 11 |
| **Available Phosphorus** | % | 0.37 | Acid-Base Balance | mEq/kg | 181 |
| **Digestible Lysine** | % | 0.749 | Sodium | % | 0.17 |
| **Digestible Methionine** | % | 0.424 | Chlorine | % | 0.21 |
| **Digestible Met + Cys** | % | 0.644 | Potassium | % | 0.65 |

**Supplementary Table 2**. Summary of each of the batches of eggs incubated and statistical analysis performed per item

| Trait | Incubation | | | | |
| --- | --- | --- | --- | --- | --- |
|  | 1 | 2 | 3 | 4 | 5 |
| Hatching eggs used | 863 | 1.455 | 733 | 723 | 5.100 |
| Breeder age (wks) | 64 | 52 | 57 | 58 | 66 |
| Saline density | Yes | Yes | No | No | No |
| Archimedes density | Yes | Yes | Yes | Yes | No |
| Eggshel, yolk and albumen weight | Yes | Yes | Yes | Yes | No |
| Yolk minerals and specific density | Yes | Yes | Yes | Yes | No |
| Geometric measurements | Yes | Yes | Yes | Yes | Yes |
| Water loss | Yes | Yes | Yes | Yes | No |
| Hatchability and embryo diagnosis | Yes | Yes | Yes | Yes | No |

**Supplementary Table 3**. Correlation matrix among the area of the eggs using the parameters of different authors and digital analysis

|  | **Area** | **Egg Area Narushin** | **Egg Area Wang et al** | **Egg Area Sauveur** | **Egg Area Besch** | **Egg Area Alkan** | **Egg Area Wang** |
| --- | --- | --- | --- | --- | --- | --- | --- |
| **Area** | – |  |  |  |  |  |  |
| **Narushin** | 0.979*** | – |  |  |  |  |  |
| **Wang et al** | 0.928*** | 0.938*** | – |  |  |  |  |
| **Sauveur** | 0.779*** | 0.807*** | 0.789*** | – |  |  |  |
| **Besch** | 0.349*** | 0.807*** | 0.789*** | 1.000*** | – |  |  |
| **Alkan** | 0.349*** | 0.807*** | 0.790*** | 1.000*** | 1.000*** | – |  |
| **Wang** | 0.977*** | 0.999*** | 0.922*** | 0.802*** | 0.802*** | 0.802*** |  |

Note. * p < 0.05, ** p < 0.01, *** p < 0.001

**Supplementary Table 4.** Correlation matrix among volume of the eggs using the parameters of different authors and digital analysis

|  | **Volume** | **Egg Volume Narushin** | **Egg Volume Wang** | **Egg Volume Alkan** | **Egg Volume KARABULUT** |
| --- | --- | --- | --- | --- | --- |
| **Volume** | – |  |  |  |  |
| **Narushin** | 0.889*** | – |  |  |  |
| **Wang** | 0.889*** | 1.000*** | – |  |  |
| **Alkan** | 0.881*** | 0.995*** | 0.995*** | – |  |
| **KARABULUT** | 0.889*** | 1.000*** | 1.000*** | 0.995*** | – |

Note. * p < 0.05, ** p < 0.01, *** p < 0.001

**Supplementary Table 5**. Number of eggs used in each analysis of specific density, weight loss, weight of the eggs, area, volume, area-to-volume ratio by image, and shape index in the eggs of each quartile analyzed

| **Quartile** | **Bottom 25%** | **Mid 25% to 50%** | **Mid 50% to 75%** | **Top 25%** |
| --- | --- | --- | --- | --- |
| **Weight (grams)** | 967 | 948 | 878 | 979 |
| **Archimedes density** | 904 | 957 | 968 | 896 |
| **Weight loss (%)** | 971 | 889 | 877 | 883 |
| **Area (cm^2^)** | 878 | 934 | 947 | 958 |
| **Volume (cm^3^)** | 871 | 946 | 947 | 948 |
| **Area:Volume Image** | 947 | 949 | 942 | 932 |
| **Shape Index** | 934 | 943 | 935 | 959 |

Bottom 25%: The eggs in the lower quartile. Mid 25% to 50%: Eggs in the quartile between 25% and 50% of the results. Mid 50% to 75%: Eggs in the quartile between 50% and 75% of the results. Top 25%: Eggs in the top 25% of the results.

**Supplementary Table 6**. Analysis of the possible isomers of the metabolites identified in the library

| **Proposed by library** | ***m/z***  **experimental** | ***Teoric m/z*** | **Error**  **ppm** | **New identification proposal** | **Molecular formula** | **Reference** |
| --- | --- | --- | --- | --- | --- | --- |
| Eudesmin  **t*_R_*** 5.836 | 409.1606 | 409.1605  [M + H]^+^ | 0.24 | Melatonin glucuronide | C_19_H_24_N_2_O_8_ | <https://hmdb.ca/metabolites/HMDB0060830> |
| Olomoucine  **t*_R_*** 6.35 | 299.1616 | 299.1619  [M + K]^+^ | -1.00 | 3,9-Dihydroxytetradecanoic acid | C_14_H_28_O_4_ | <https://hmdb.ca/metabolites/HMDB0340783> |
|  | 299.1616 | 299.1619  [M + K]^+^ | -1.00 | 3,7-Dihydroxytetradecanoic acid | C_14_H_28_O_4_ | <https://hmdb.ca/metabolites/HMDB0340775> |
|  | 299.1616 | 299.1619  [M + K]^+^ | -1.00 | 3,10-Dihydroxytetradecanoic acid | C_14_H_28_O_4_ | <https://hmdb.ca/metabolites/HMDB0340780> |
|  | 299.1616 | 299.1619  [M + K]^+^ | -1.00 | 3,4-Dihydroxytetradecanoic acid | C_14_H_28_O_4_ | <https://hmdb.ca/metabolites/HMDB0340778> |
|  | 299.1616 | 299.1619  [M + K]^+^ | -1.00 | 3,13-Dihydroxytetradecanoic acid | C_14_H_28_O_4_ | <https://hmdb.ca/metabolites/HMDB0340776> |
|  | 299.1616 | 299.1619  [M + K]^+^ | -1.00 | 3,11-Dihydroxytetradecanoic acid | C_14_H_28_O_4_ | <https://hmdb.ca/metabolites/HMDB0340776> |

**
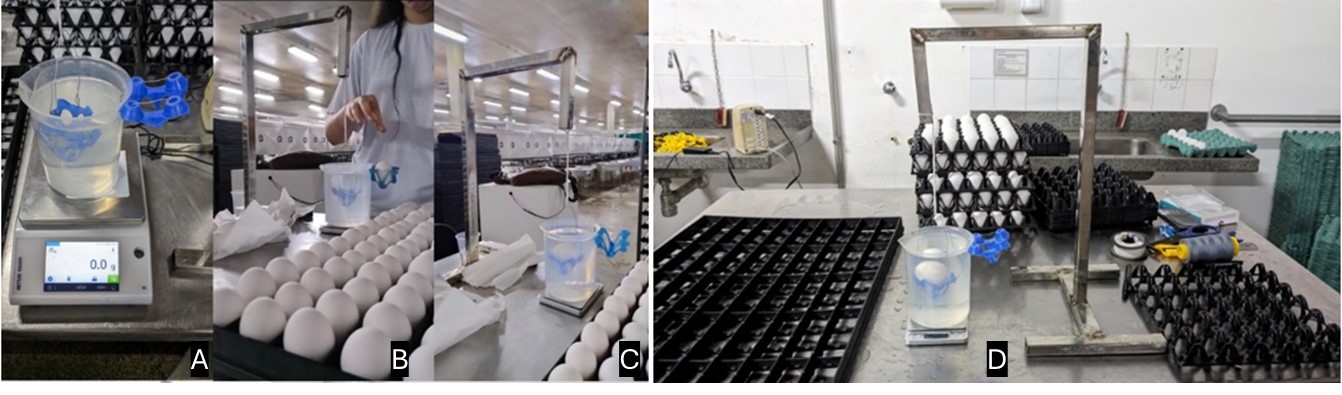
Supplementary Figure 1.** Apparatus built for individual egg density measurement according to the Archimedes principle

**A**: tared digital scale with water and plastic cup for weighing immersed and dry egg weight; **B**: Weighing of egg outside water; **C**: Weighing of the egg immersed in water; **D**: the whole set up made for measuring egg density by the Archimedes principle.

**Supplementary Figure 2.** Image capture and processing system of the geometric shape of the eggs

**
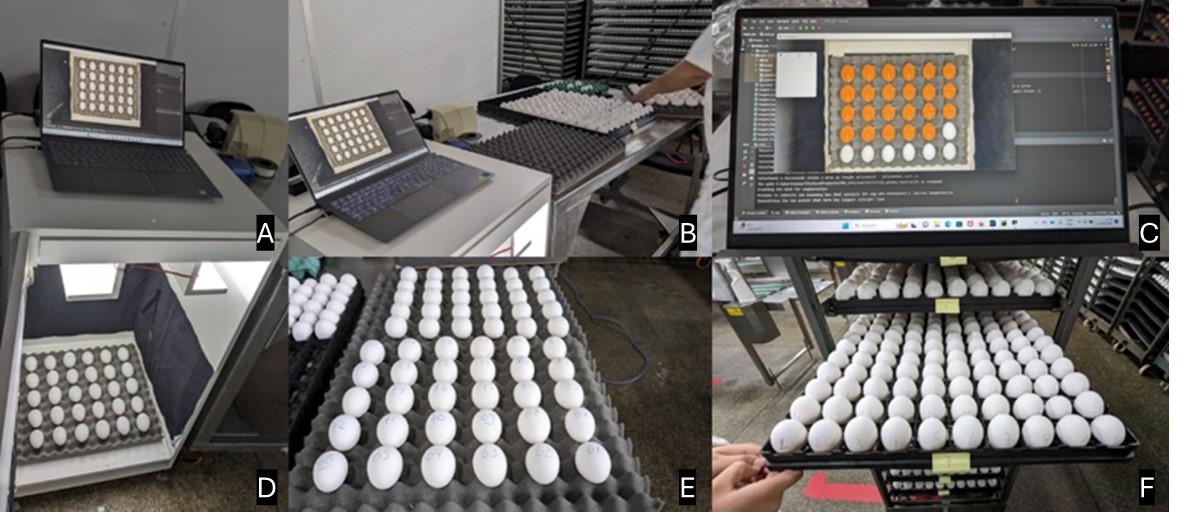
**

**A**: computer showing scanned images; **B**: computer with scanned images and trayed eggs waiting for capturing; **C**: Ongoing of scanning process egg by egg; **D**: Eggs displayed on the cabinet/chamber ready for image capturing; **E**: 30-egg tray set up for image capturing; **F**: eggs and trays identified after completion of image capturing
